# Supplementary material for: Evolution of an extreme hemoglobin phenotype contributed to the sub-Arctic specialization of extinct Steller’s sea cows
Source: eLife. 2023 Jun 1;12:e85414. doi: 10.7554/eLife.85414 (PMC10284601; doi:10.7554/eLife.85414)
Supplement: Supplementary file 1. — (a) Intrinsic oxygen affinities (P50, mmHg) for the adult-expressed hemoglobins (α2β/δ2) of the Florida manatee (Trichechus manatus latirostris), dugong (Dugong dugon), Steller’s sea cow (Hydrodamalis gigas), and the ancestral dugongid (‘Anc. dugongid’), and their sensitivity to allosteric effectors at 25 and 37°C in 0.1 M HEPES buffer. All values are corrected to pH 7.2. (b) Intrinsic oxygen affinities (P50, mmHg) for the prenatally-expressed hemoglobins Gower I (ζ2ε2) and HbF (α2γ2) of Steller’s sea cow (Hydrodamalis gigas) and HbF (α2γ2) of the dugong (Dugong dugon), and their sensitivity to allosteric effectors at 37°C in 0.1 M HEPES buffer. All values are corrected to pH 7.1 to account for the lower pH of prenatal blood. (c) Accession numbers for beta-globin amino acid sequences used for ConSurf analyses. [file elife-85414-supp1.docx]

**Supplementary File 1 for**

Evolution of an extreme hemoglobin phenotype contributed to the sub-Arctic specialization of extinct Steller's sea cows.

Anthony V. Signore^1†^, Phillip R. Morrison^2‡^, Colin J. Brauner^2^, Angela Fago^3^, Roy E. Weber^3^ and Kevin L. Campbell^1^*

^1^Department of Biological Sciences, University of Manitoba, Winnipeg, R3T 3N2, Canada

^2^Department of Zoology, University of British Columbia, Vancouver, V6T 1Z4, Canada

^3^Department of Biology, Zoophysiology, Aarhus University, DK-8000 Aarhus, Denmark

***For correspondence:** Kevin.Campbell@umanitoba.ca

**Present address:** ^†^National Centre for Foreign Animal Disease, Winnipeg, R3E 3M4, Canada; ^‡^Department of Resource Management and Protection, and Biology Department, Vancouver Island University, Nanaimo, V9R 5S5, Canada

**This file includes:**

Supplementary File 1a

Supplementary File 1b

Supplementary File 1c

Supplementary File 1a. Intrinsic oxygen affinities (P_50_, mmHg) for the adult-expressed hemoglobins (α_2_β/δ_2_) of the Florida manatee (*Trichechus manatus latirostris*), dugong (*Dugong dugon*), Steller’s sea cow (*Hydrodamalis gigas*), and the ancestral dugongid (‘Anc. dugongid’), and their sensitivity to allosteric effectors at 25 and 37°C in 0.1 M HEPES buffer. All values are corrected to pH 7.2.

| **Species** | **Temp** | ^a^**P_50_** | ^a^**logP_50_** | ^b^**Cl^-^ effect** | ^c^**DPG effect** | ^d^Bohr effect (ΔlogP_50_/ΔpH) | | | |
| --- | --- | --- | --- | --- | --- | --- | --- | --- | --- |
|  |  |  |  |  |  | **Stripped** | **+KCl** | **+DPG** | **KCl + DPG** |
| *T. manatus* | 37°C | 5.36 | 0.73 | 0.23 | 0.14 | -0.14 | -0.28 | -0.39 | -0.34 |
|  | 25°C | 1.88 | 0.27 | 0.30 | 0.29 | -0.14 | -0.28 | -0.59 | -0.42 |
| *Anc. dugongid* | 37°C | 4.30 | 0.63 | 0.29 | 0.27 | -0.12 | -0.26 | -0.36 | -0.60 |
|  | 25°C | 1.86 | 0.27 | 0.35 | 0.40 | -0.21 | -0.34 | -0.67 | -0.61 |
| *D. dugon* | 37°C | 3.51 | 0.55 | 0.39 | 0.30 | -0.13 | -0.35 | -0.58 | -0.36 |
|  | 25°C | 1.29 | 0.11 | 0.42 | 0.39 | -0.21 | -0.34 | -0.68 | -0.49 |
| *H. gigas* | 37°C | 8.80 | 0.94 | 0.17 | 0.02 | -0.15 | -0.25 | -0.13 | -0.27 |
|  | 25°C | 4.25 | 0.63 | 0.24 | 0.03 | -0.19 | -0.32 | -0.30 | -0.42 |
| *H. gigas* β82k | 37°C | 5.39 | 0.71 | 0.25 | 0.27 | -0.41 | -0.32 | -0.36 | -0.46 |
|  | 25°C | 1.72 | 0.23 | – | – | -0.14 | – | – | -0.65 |

^a^PO_2_ at half-saturation (mm Hg)

^b^ΔlogP_50_^(KCl – stripped)^

^c^ΔlogP_50_^(DPG – stripped)^

^d^pH range 6.9 to 7.8

Supplementary File 1b. Intrinsic oxygen affinities (P_50_, mmHg) for the prenatally-expressed hemoglobins Gower I (ζ_2_ε_2_) and HbF (α_2_γ_2_) of Steller’s sea cow (*Hydrodamalis gigas*) and HbF (α_2_γ_2_) of the dugong (*Dugong dugon*), and their sensitivity to allosteric effectors at 37°C in 0.1 M HEPES buffer. All values are corrected to pH 7.1 to account for the lower pH of prenatal blood.

| **Hb** | ^a^**P_50_** | ^a^**logP_50_** | ^b^**Cl^-^ effect** | ^c^**DPG effect** | ^d^Bohr effect (ΔlogP_50_/ΔpH) | | | |
| --- | --- | --- | --- | --- | --- | --- | --- | --- |
|  |  |  |  |  | **Stripped** | **+KCl** | **+DPG** | **KCl + DPG** |
| *H. gigas* Gower I | 1.90 | 0.28 | 0.07 | 0.23 | 0.18 | -0.05 | -0.21 | 0.20 |
| *H. gigas* HbF | 0.53 | -0.27 | -0.15 | 0.13 | 0.07 | -0.50 | 0.58 | -0.09 |
| *D. dugon* HbF | 0.57 | -0.24 | 0.40 | 0.20 | -0.10 | 0.68 | 0.56 | 0.16 |

^a^PO_2_ at half-saturation (mm Hg)

^b^ΔlogP_50_^(KCl – stripped)^

^c^ΔlogP_50_^(DPG – stripped)^

^d^pH range 6.75 to 7.15

Supplementary File 1c. Accession numbers for beta-globin amino acid sequences used for ConSurf analyses.

| **Organism** | **Common name** | **Accession #** |
| --- | --- | --- |
| *Alces alces alces* | European elk | P02073 |
| *Ammotragus lervia* | Aoudad | ABC86528 |
| *Ateles geoffroyi* | Black-handed spider monkey | P68232 |
| *Balaenoptera acutorostrata* | Minke whale | P18984 |
| *Bison bonasus* | European bison | P09422 |
| *Bos taurus* | Cattle | P02070 |
| *Bradypus tridactylus* | Pale-throated sloth | AAZ22685 |
| *Bubalus bubalis* | Water buffalo | P67820 |
| *Camelus bactrianus* | Bactrian camel | P68230 |
| *Canis lupus familiaris* | Domestic Dog | P60524 |
| *Capra hircus* | Goat | P02077 |
| *Cavia porcellus* | Domestic guinea pig | P02095 |
| *Ceratotherium simum* | White rhinoceros | P02066 |
| *Dasypus novemcinctus* | Nine-banded armadillo | AAZ22684 |
| *Didelphis virginiana* | North American opossum | P02109 |
| *Dugong dugon* | Dugong | QBK14998 |
| *Echinops telfairi* | Small Madagascar hedgehog | AAB21591 |
| *Elephas maximus* | Asiatic elephant | ACV41403 |
| *Equus caballus* | Horse | P02062 |
| *Erinaceus europaeus* | Western European hedgehog | P02059 |
| *Eulemur fulvus fulvus* | Common brown lemur | P02053 |
| *Felis catus* | Domestic cat | P07412 |
| *Hippopotamus amphibius* | Hippopotamus | P19016 |
| *Homo sapiens* | Human | P68871 |
| *Lama glama* | Llama | P68226 |
| *Loxodonta africana* | African savanna elephant | ACV41399 |
| *Macaca mulatta* | Rhesus monkey | P02026 |
| *Macropus eugenii* | Tammar wallaby | Q6H1U7 |
| *Macropus giganteus* | Eastern gray kangaroo | P02106 |
| *Mammuthus primigenius* | Woolly mammoth | ACV41408 |
| *Myotis velifer* | Mouse-eared bat | P11758 |
| *Odocoileus virginianus virginianus* | Virginia white-tailed deer | P02074 |
| *Ornithorhynchus anatinus* | Platypus | P02111 |
| *Oryctolagus cuniculus* | Rabbit | P02057 |
| *Ovis aries musimon* | European mouflon | P02076 |
| *Procavia capensis* | Cape rock hyrax | Q45XI6 |
| *Rangifer tarandus* | Reindeer | P21380 |
| *Rattus norvegicus* | Norway rat | P02091 |
| *Rousettus aegyptiacus* | Egyptian rousette | P02058 |
| *Suncus murinus* | House shrew | P02060 |
| *Sus scrofa* | Pig | P02067 |
| *Tachyglossus aculeatus aculeatus* | Short-beaked echidna | P02110 |
| *Talpa europaea* | European mole | P02061 |
| *Tapirus terrestris* | Brazilian tapir | P02064 |
| *Tarsius bancanus* | Horsfield's tarsier | P02051 |
| *Tragelaphus strepsiceros* | Greater kudu | P04245 |
| *Trichechus manatus* | West Indian manatee | Q45XI8 |
| *Tupaia glis* | Common tree shrew | P02052 |
| *Tursiops truncatus* | Bottlenose dolphin | P18990 |
| *Ursus maritimus* | Polar bear | P68011 |
| *Varecia variegata* | Ruffed lemur | P21667 |
